# Narrative Experiences of Esketamine-Induced Dissociation in Patients with Treatment-Resistant Depression: A Qualitative Exploratory Study

**DOI:** 10.3390/brainsci16020196

**Published:** 2026-02-07

**Authors:** Miriam Olivola, Tiziano Prodi, Giada Versaci, Chiara Angeletti, Kevin La Monica, Fabiola Raffone, Nicolaja Girone, Natascia Brondino, Roberta Anniverno, Vassilis Martiadis, Giovanni Martinotti, Bernardo Dell’Osso

**Affiliations:** 1 Department of Brain and Behavioral Sciences, University of Pavia, 27100 Pavia, Italy; miriam.olivola@asst-fbf-sacco.it (M.O.);; 2 Department of Mental Health and Addiction Services, ASST Fatebenefratelli-Sacco, 20157 Milan, Italy; 3 Department of Mental Health, Department of Biomedical and Clinical Sciences “Luigi Sacco”, University of Milan, 20157 Milan, Italy; 4 Macedonio Melloni Hospital, ASST Fatebenefratelli-Sacco, 20129 Milan, Italy; 5 Department of Mental Health, ASL Napoli 1 Centro, Community Mental Health Center DS 25, 80125 Naples, Italy; 6 Department of Neuroscience, Imaging and Clinical Sciences, “G. D’Annunzio” University, 66100 Chieti, Italy; 7 “Aldo Ravelli” Center for Nanotechnology and Neurostimulation, University of Milan, 20157 Milan, Italy; 8 Department of Psychiatry and Behavioral Sciences, Stanford University, Stanford, CA 94305, USA; 9 Centro per lo Studio dei Meccanismi Molecolari alla Base delle Patologie Neuro-Psico-Geriatriche, University of Milan, 20157 Milan, Italy

**Keywords:** esketamine, dissociation, treatment-resistant depression, narrative medicine, altered states of consciousness, phenomenology, qualitative psychiatry, dissociative experience

## Abstract

**Background/Objectives**: Esketamine-related dissociation is a transient, pharmacologically induced altered state that differs from the trait-like pathological dissociation typically observed in trauma-related conditions. While most studies have quantified these effects using the Clinician-Administered Dissociative States Scale (CADSS), patients’ subjective phenomenology and meaning-making remain underexplored. This qualitative exploratory study investigated how patients narrate, interpret, and integrate dissociative experiences occurring during intranasal esketamine treatment for treatment-resistant depression (TRD). **Methods**: We conducted semi-structured interviews with 36 adults with TRD who were receiving intranasal esketamine in outpatient settings in Northern Italy (2022–2024). Interviews focused on the most salient dissociative experiences during treatment. Transcripts were anonymized and analyzed using inductive thematic analysis. Two researchers coded the data independently; discrepancies were resolved by consensus, and recruitment continued until thematic saturation was reached. **Results**: Four experiential domains emerged: sensory alteration and perceptual flow (10/36, 27.8%), time suspension and chronological drift (21/36, 58.3%), body and space alteration (20/36, 55.6%), and psychic distance from suffering (30/36, 83.3%). While a minority described transient distress or loss of control, most narratives framed dissociation as neutral or subjectively meaningful, often associated with a temporary reduction in ruminative self-focus and depressive distress. **Conclusions**: A narrative, phenomenological lens complements quantitative research by clarifying what esketamine-induced dissociation feels like to patients and how it is appraised in context. The findings do not imply a causal or mediating role in antidepressant efficacy. Rather, they suggest that dissociation functions as a transitional subjective state, the clinical relevance of which depends on anticipation, framing, monitoring, and integration. These results support the use of structured psychoeducation, in-session support, and post-session integration within real-world esketamine programs.

## 1. Introduction

Dissociation is increasingly recognized as a multidimensional phenomenon involving disruptions in the integrated functioning of consciousness, perception, memory, and identity [1,2]. More specifically, it can be described as a disconnection between a person’s thoughts, memories, feelings, actions, or sense of self. Rather than representing a unitary construct, dissociation encompasses a continuum ranging from mild, transient states of detachment—such as absorption or daydream-like experiences—to clinically impairing syndromes characterized by fragmentation of identity and loss of continuity in self-experience [2]. Several phenomenological subtypes have been consistently identified within this spectrum, reflecting the heterogeneity of dissociative experiences [2].

It is important to note that the phenomenology and clinical significance of dissociation can vary substantially depending on its etiology and contextual embedding. In psychopathological conditions, dissociation is typically pathological or trait-like, occurring in trauma-related disorders, panic states, or psychotic conditions. In these contexts, depersonalization often manifests as a distressing sense of estrangement from one’s own body or thoughts, while derealization involves a pervasive alteration in the perception of external reality [3]. Such experiences are often accompanied by anxiety, fear, and impaired reality testing. More severe manifestations, including dissociative amnesia or identity fragmentation, are most commonly observed in trauma-related disorders such as dissociative identity disorder.

By contrast, pharmacologically induced dissociation—as observed with dissociative agents such as ketamine and esketamine, and more broadly with psychedelic compounds—is typically transient, reversible, and not necessarily pathological. Patients often report perceptual intensification, altered embodiment, or dream-like states. When these occur in a controlled therapeutic context, they are frequently described as neutral or non-distressing. These drug-induced experiences more closely resemble altered states of consciousness (ASC), which are commonly assessed in psychedelic research using instruments such as the Altered States of Consciousness Rating Scale (ASC-5) [4], than the pathological dissociation captured by trauma-oriented measures such as the CADSS [5]. Nonetheless, the CADSS remains the most widely used instrument in esketamine trials, raising important questions about whether current measurement tools adequately capture the lived experience of pharmacologically induced dissociation. Notably, emerging phenomenological clinical work has described disembodiment and affective resonances during intranasal esketamine in patients presenting a depersonalized depression subtype [6].

The emergence of dissociative experiences during esketamine administration has generated considerable debate: should dissociation be regarded merely as a pharmacological epiphenomenon, or might it play a functional role in therapeutic response? Early ketamine studies suggested modest associations between dissociative intensity and short-term antidepressant outcomes, but mediation analyses did not support dissociation as the mechanism of symptom improvement [7]. In the specific context of intranasal esketamine, post hoc analyses of randomized trials have reported no clinically meaningful association between dissociation severity and antidepressant response, nor any consistent mediation of symptom change by dissociative effects [8,9]. These findings align with conceptual accounts that interpret dissociation as an acute altered state relevant for clinical preparation, monitoring, and integration, rather than as an indicator of efficacy [10,11].

Consistent with this perspective, converging evidence suggests that esketamine-related dissociation is not a reliable indicator of antidepressant efficacy. Post hoc analyses of phase 3 trials revealed that dissociative and antidepressant effects were not significantly correlated, and dissociation did not mediate symptom improvement or relapse prevention [8]. Similarly, a pooled analysis of TRANSFORM-1 and TRANSFORM-2 studies found no clinically meaningful association between peak dissociation (CADSS) and MADRS change during the four-week induction phase [9]. These data support interpreting dissociation as a common acute subjective effect that is clinically relevant for preparation and monitoring, rather than as a marker of response.

Despite extensive quantitative investigation, relatively little is known about how patients subjectively experience, interpret, and integrate dissociative states into their personal narratives of illness and recovery. The present study addresses this gap by moving beyond correlational analyses to explore the lived experience of dissociation as reported by patients undergoing intranasal esketamine treatment for TRD. Drawing on a curated sample of 36 detailed patient narratives collected in real-world clinical settings in northern Italy between 2022 and 2024, we examine dissociation as a transitional experiential state rather than a mechanism of efficacy. Through inductive narrative analysis, we aim to deepen our understanding of how patients perceive, endure, and make sense of these altered states, adopting a phenomenological approach rather than imposing predefined diagnostic or mechanistic frameworks.

The primary aim of this qualitative exploratory study was to investigate the subjective meaning of dissociative experiences within routine clinical practice during intranasal esketamine treatment for TRD. Accordingly, the study does not aim to evaluate the clinical efficacy of esketamine or to estimate remission rates. Specifically, we asked: which experiential domains are most salient during intranasal esketamine sessions; how patients appraise these experiences as helpful, neutral, or distressing; and which contextual elements (anticipation, setting, support, and integration) shape meaning-making. This focus is intended to inform patient-centered psychoeducation and clinical monitoring in real-world esketamine programs.

## 2. Materials and Methods

### 2.1. Study Design and Setting

We conducted a qualitative study involving 36 consecutive patients recruited from outpatient psychiatric centers in ASST Pavia and ASST Fatebenefratelli Sacco Milan who were undergoing intranasal esketamine treatment for TRD between 2022 and 2024.

### 2.2. Participants

Inclusion criteria included: diagnosis of Major Depressive Disorder (MDD) according to DSM-5 criteria; treatment resistance, defined as non-response to at least two adequate antidepressant trials involving different antidepressant molecules; eligibility for intranasal esketamine according to EMA prescribing guidelines, including an absence of contraindications; completion of at least two esketamine treatment sessions prior to the interview; capacity to provide informed consent. Exclusion criteria were: a current or past diagnosis of a primary psychotic disorder, bipolar I disorder, or borderline personality disorder with prominent dissociative features; substance use disorder within the previous 6 months (excluding nicotine and caffeine); severe or uncontrolled medical conditions (e.g., cardiovascular or thyroid conditions) that contraindicated esketamine treatment; pregnancy or breastfeeding; severe cognitive impairment or an inability to participate in a narrative interview.

### 2.3. Interview Procedure and Data Collection

Data were collected through semi-structured interviews conducted within one week after an esketamine administration session. Patients were invited to retrospectively describe their most significant dissociative experience during treatment. Guiding prompts included: “Can you describe in detail what you experienced during the session?”, “How did your perception of time, space, or your body change?”, and “How did the experience affect your emotional state or your relationship with depressive symptoms?”. The interviews were audio-recorded, transcribed verbatim, and anonymized. Reporting was guided by the Consolidated Criteria for Reporting Qualitative Research (COREQ) framework [12]. The interviews were conducted in Italian by a trained psychiatrist, who was not involved in the routine clinical care of participants. The interviews took place in a private outpatient setting and lasted approximately 45 min. Participants were informed of this to reduce social desirability and encourage open narration.

### 2.4. Qualitative Analysis

Narratives were analyzed using an inductive thematic analysis following Braun and Clarke’s six-phase framework [13]. Coding was performed independently by two researchers using NVivo software (Lumivero, version 12), with discrepancies resolved through consensus meetings with a senior investigator. Decisions about coding and theme development were documented iteratively across meetings, providing an audit trail of analytical choices. Data saturation was reached when no new themes emerged from consecutive interviews, as jointly assessed by the coding team during the iterative analysis. A topic guide was used to ensure coverage of key experiential dimensions while allowing free narration [14]; the guide is available from the authors upon reasonable request. For publication, quotations were translated into English by bilingual investigators and checked for semantic equivalence.

### 2.5. Assessment and Quantitative Measures

Narrative analysis does not aim to replace standardized dissociation scales, but rather to address a different epistemological level. While instruments such as the CADSS quantify dissociative intensity, narrative methods explore meaning, appraisal and integration, dimensions that are not captured by symptom severity scores. Standardized dissociation rating scales (e.g., CADSS) were not prospectively collected within this qualitative protocol, and baseline trait dissociation/trauma history was not systematically characterized. Therefore, we report thematic prevalence descriptively and did not conduct inferential statistical testing linking dissociative intensity to clinical outcomes.

### 2.6. Ethics

All study procedures adhered to the principles of Good Clinical Practice (GCP) and the principles of the Declaration of Helsinki [15]. Ethical approval was obtained from the Pavia Ethics Committee (Opinion No. 84157/21, 27 August 2021; amendment No. 0102231/21). Written informed consent was obtained from all participants following a comprehensive explanation of the study’s aims and procedures. Audio files and transcripts were stored securely in accordance with institutional policies, and all identifying information was removed during transcription. Data are available upon reasonable request.

## 3. Results

The following results are derived from a qualitative narrative analysis conducted using NVivo 12. Four overarching experiential themes were identified: sensory alteration and perceptual flow, time suspension and chronological drift, body and space alteration, and psychic distance from suffering. Table 1 summarizes these themes and the proportion of patients who reported them. Across all themes, only a small proportion of patients reported transient distress (e.g., fear, disorientation, or loss of control), which was typically short-lived and alleviated by reassurance and grounding in the clinical setting.

### 3.1. Participant Characteristics

A descriptive table of the sample is provided (Table 2), summarizing age and key depression-related clinical characteristics (e.g., age, age at first MDE, number of psychiatric comorbidities, and medication exposure as extracted from clinical records).

### 3.2. Sensory Alteration and Perceptual Flow

*“Colors weren’t just brighter—they were conscious. The room breathed with me.” “It was as if I was in a cloud, floating through tunnels of bright purple, and it felt as if I was floating inside the entire world”*. Ten participants (27.8%) reported profound alterations in sensory perception, particularly in the visual domain. Dissociation began with a change in how the world looked and felt. One patient described the walls *“melted into rivers of color,”* while another spoke of *“touching sound” *and *“hearing light”*. A participant noted, *“My hands looked like liquid. I waved them in front of me and they left trails, like comets in slow motion”.* Several patients mentioned a sense of synesthesia, in which boundaries between the senses became permeable: *“Voices had texture. My therapist’s voice felt like velvet on my skin”*. The same group of participants (n = 10) also described auditory changes, which often occurred alongside visual alterations. The ticking of the clock became a deep, reverberating drum. Everyday background noise, such as footsteps, doors, and human speech, was often described as distant, slowed, or transformed into an ambient hum. Another participant said, *“I heard silence for the first time in my life. It was enormous”*.

### 3.3. Time Suspension and Chronological Drift

*“Time didn’t move forward. It spiralled”*. Twenty-one participants (58.3%) described marked alterations in their perception of time. Many experienced time dilation, perceiving that minutes extended into hours: *“I had entire conversations in my mind that must have taken hours. When I came back, only ten minutes had passed”.* Several reported time loops, in which sensations seemed to reset in repetitive cycles, evoking déjà vu: *“It felt like being stuck in a single breath that kept resetting”*. While some found this collapse of temporal continuity disorienting, others interpreted it positively: *“There was no past, no future, just the present moment, and that was enough”*. *“I didn’t know if I had been there for seconds or forever”*, admitted one patient.

### 3.4. Body and Space Alteration

*“I felt like a soap bubble—delicate, translucent, and no longer tied to the ground”.* Twenty participants (55.6%) reported alterations in embodiment and spatial perception. Within this theme, many patients described a sense of detachment from their physical body, most commonly experiencing a sensationof floating or weightlessness. A smaller subset described more radical disruptions, including out-of-body experiences or spatial dislocation. One patient recalled: *“I saw myself curled up in the chair, small and soft. I felt compassion for that person—it was me, but not quite”*. Several participants reported a dissolution of boundaries between the self and the environment, expressing a sense of merging with their surroundings: *“There was no more me versus the world. I was the room, the sound, the pulse of everything”*. Another remarked: *“My body dissolved into space. I wasn’t in the room—I was the room”*.

### 3.5. Psychic Distance from Suffering

Thirty participants (83.3%) reported a temporary yet profound shift in their relationship with emotional pain. For many, depressive thoughts remained present but were experienced as remote and less intrusive. One participant, who had experienced chronic suicidal thoughts for a long time, explained: *“The thoughts were still there, but they were like distant birds flying behind glass. They couldn’t get to me”*. Others described unexpected moments of relief, often framed as a suspension of self-critical rumination: *“For one hour, my inner critic went silent. I didn’t realize how loud it had been until it was gone.”* Several patients referred to this experience as a *“pause” *from the relentless cycle of guilt, hopelessness, and self-reproach that they otherwise experienced: *“It felt like putting my sadness down for a moment. Like setting down a heavy bag you’ve carried for too long”*. Narratives frequently employed metaphors of distance and separation, such as *“The pain was in the next room” *or *“I could see the darkness, but I wasn’t in it”*. One participant summarized their experience as follows: *“I finally stepped outside the storm”*. Although these reprieves were often transient, they were perceived as pivotal. Patients emphasized that even a brief interruption to suffering could be transformative: *“That break—just those few minutes—showed me that relief is possible. It gave me a reason to keep going”*. Although these alterations were profound, they were rarely perceived as distressing. On the contrary, most patients interpreted them as peaceful, expansive, or emotionally neutral.

## 4. Discussion

While four experiential domains were identified (see Table 1), the following discussion first highlights the phenomenological motifs most salient in patients’ narratives and then situates them within existing neurocognitive accounts of ketamine/esketamine-induced altered states. Because we did not collect neuroimaging or physiological data, these links are intended as conceptual and hypothesis-generating contextualization, rather than evidence of causal mechanisms [10,11].

Firstly, the attenuation of self-referential thought described by patients as “being outside myself” or “escaping my own mind” can be interpreted as an experiential correlate of a temporary loosening of rigid self-focused processing during the acute state [10,11]. Secondly, reductions in prefrontal cortical activity, as discussed in neurobiological accounts of the acute ketamine/esketamine state, may provide a neurobiological context for patients’ descriptions of “*silence in my thoughts*” or “*switching off*”. At the subjective level, this state was frequently experienced as a suspension of excessive top-down cognitive control, allowing a temporary disengagement from compulsive overprocessing. Some participants have described this process metaphorically as “*stepping out of the machinery*”. Thirdly, several narratives converged on the emergence of a present-centered mode of awareness. Expressions such as “*just being here*” or “*everything was now*” suggest that, for some individuals, dissociative experiences were accompanied by a shift away from ruminative self-states towards an immediate experiential focus. Notably, this phenomenological quality resembles aspects of spontaneous mindfulness or flow, without implying intentional or sustained attentional training. In addition, alterations in temporal experience and body–space perception were frequently reported, ranging from time dilation and temporal discontinuity to feelings of bodily lightness, detachment, or spatial expansion. These accounts indicate that esketamine-induced dissociation is not a unitary phenomenon and highlight the importance of anticipatory framing and individualized support, particularly when patients describe destabilizing or unfamiliar sensations related to their bodies [16,17]. Taken together, these themes suggest that esketamine-induced dissociation can be interpreted as an acute, time-limited shift in self-processing that may temporarily loosen the coupling between affective distress and the habitual narrative self. From this perspective, the frequently reported ‘psychic distance from suffering’ can be framed as a state-level emotion-regulation phenomenon (i.e., a form of experiential decentering) rather than as a mere adverse event, even though it is neither necessary nor sufficient for antidepressant response [3,10,11]. Importantly, both the subjective quality and the appraisal of this altered state are likely to be shaped by individual differences—including baseline dissociative tendencies, trauma exposure, emotional sensitivity, and prior relational experiences—which we did not assess systematically and therefore cannot examine as moderators within the present dataset [16,17]

Consistent with randomized-trial evidence, the intensity of esketamine-induced dissociation does not reliably predict antidepressant response, and dissociation has not been shown to be a mechanism mediating symptom improvement or relapse prevention [18,19,20]. Taken together, these findings challenge the simplistic framing of dissociation as merely an adverse event or pharmacological side effect. Instead, dissociative phenomenology appears to represent a clinically relevant, time-limited subjective state that should be anticipated, contained, and integrated within routine care. It should not be used as an indicator of antidepressant efficacy. While uncontained or distressing experiences may present clinical challenges, the narratives reported here suggest that, within an adequately supportive context, dissociation is frequently perceived as neutral and, in certain instances, as meaningful or beneficial. Accordingly, this study does not claim mechanistic insight; instead, it offers a phenomenological account of how patients interpret esketamine-related altered experiences and how contextual factors may shape their appraisal, without implying a causal role in reducing symptoms [8,9].

### 4.1. Clinical Implications

The highly subjective and context-dependent nature of dissociative experiences highlights the importance of careful clinical management. While most participants described dissociation as tolerable or occasionally beneficial, some reported distressing reactions, such as fear, disorientation, or existential discomfort. Notably, these responses were often linked to inadequate anticipatory preparation rather than the intensity of dissociation itself [21,22,23,24,25]. From a clinical perspective, three key implications can be derived from these findings. First, pre-treatment psychoeducation and a clear initial explanation of possible dissociative phenomena appear essential. Providing patients with clear, balanced, and non-alarmist information about the possible phenomenology of dissociation may reduce anticipatory anxiety and encourage acceptance of the experience. Secondly, in-session monitoring is crucial. In real-world settings, outcomes beyond depressive symptom reduction, such as suicidal and self-harming responses, may demonstrate clinically relevant heterogeneity, including gender-related patterns reported during esketamine treatment [26,27]. In this context, recent meta-analytic work has also highlighted remaining uncertainties regarding the long-term efficacy and safety of intranasal esketamine, reinforcing the importance of structured explanation, in-session support, and monitoring to optimize tolerability and support safe implementation in routine care [28]. Clinicians should pay close attention to signs of psychological distress, especially in individuals with a history of trauma or an increased risk of experiencing psychotic symptoms, and be ready to provide reassurance or grounding techniques when necessary. Thirdly, post-session integration may represent a valuable component of clinical care. Even brief opportunities for reflective dialog can help patients to contextualize their experiences, facilitating meaning-making and reducing the likelihood that dissociative episodes will be perceived as frightening or destabilizing. These practices support a non-pathologizing, phenomenologically informed approach, in which dissociation is explored in terms of its subjective qualities, as well as in relation to what it temporarily disrupts and enables at the experiential level.

### 4.2. Limitations

Several limitations should be acknowledged. The sample size and the restricted geographical recruitment may limit the transferability to other healthcare systems, cultures, and treatment settings (e.g., inpatient programs or different esketamine delivery models). Furthermore, interviews took place after at least two sessions and within one week of an administration; individuals who discontinued early, experienced severe distress, or were less willing to reflect on the experience may be underrepresented, which could introduce potential selection and survivorship bias. Thirdly, the study relied on retrospective narratives, which are inherently shaped by memory, current mood state, and post hoc meaning-making; therefore, some accounts may reflect reconstructed rather than reflecting “in-the-moment” phenomenology. Fourthly, we did not systematically characterize baseline dissociative traits, depersonalization/derealization severity, or trauma history, which could plausibly modulate the content and appraisal of dissociative experiences. Relatedly, we did not assess developmental or relational variables (e.g., attachment-relevant histories) that shape emotion/feeling processes and self-processing trajectories and may influence how altered states are appraised and integrated; this represents an additional interpretive limitation and a priority for future research [29]. Fifthly, as with any interpretive thematic analysis, themes may partly reflect the researchers’ perspectives and the clinical context in which the interviews took place; social desirability and the interviewer–participant relationship may have influenced the responses, and translating the selected quotations into English may have reduced the nuances. Finally, given the qualitative design, limited size, and the absence of a control group, any descriptive response rates reported in this sample should not be interpreted as estimates of esketamine efficacy.

## 5. Conclusions

Esketamine-induced dissociation is commonly described as a transient shift in self-perception, often involving reduced rumination and greater psychological distance from suffering. In this qualitative sample, dissociation was generally viewed as neutral or meaningful when adequately anticipated and supported. These findings support integrating structured psychoeducation, in-session monitoring and grounding, and brief post-session integration into routine esketamine programs to optimize tolerability and support safe implementation, while avoiding the use of dissociative intensity as an efficacy marker. This emphasis is particularly relevant in light of recent findings, which discuss ongoing questions regarding long-term efficacy and safety. Future research should combine prospective, in-the-moment assessments of dissociative phenomenology with standardized symptom and safety outcomes, and test how preparation, setting, and post-session integration shape appraisal and tolerability in different clinical contexts.

## Figures and Tables

**Table 1:** Main experiential themes of esketamine-induced dissociation (N = 36).

Theme	Patients Reporting, n (%)	Representative Quotation
Sensory alteration and perceptual flow	10 (27.8%)	*My hands looked like liquid. I waved them * *in front of me and they left trails, * *like comets in slow motion*
Time suspension and chronological drift	21 (58.3%)	*It felt like being stuck in a single breath * *that kept resetting*
Body and space alteration	20 (55.6%)	*There was no more me versus the world. I was the room, the sound, the pulse of everything.*
Psychic distance from suffering	30 (83.3%)	*The thoughts were still there—but they were like distant birds, flying behind glass*

**Table 2:** Clinical features of the sample.

Variable	n	Mean	Min	Max	SD
Age (years)	36	45.94	19	79	18.00
Age at First MDE (years)	36	30.83	12	79	15.32
Concomitant Antidepressants at baseline (n)	36	1.08	1	3	1.13
Number of Previous Antipsychotics (n)	36	1.28	0	6	1.37
Number of Comorbidities (n)	36	1.64	0	5	1.33
Current MDE Duration (months)	36	17.34	3	84	15.49
Number of Previous MDEs (n)	36	4.78	0	40	6.71
Number of lifetime suicide attempts (n)	36	0.72	0	3	0.97

## Data Availability

Data available on reasonable requests. The data are restricted due to patient privacy/confidentiality reasons.

## Abbreviations

The following abbreviations are used in this manuscript:

CADSS	Clinician-Administered Dissociative States Scale
TRD	Treatment-resistant depression
ASC	Altered States of Consciousness Scale
MADRS	Montgomery–Asberg Depression Rating Scale
MDD	Major depressive disorder
DSM-5	Diagnostic and statistical manual of mental disorders–5
EMA	European Medicines Agency
COREQ	Consolidated criteria for reporting qualitative medicine
GCP	Good clinical practice
DMN	Default mode network
